# Direct theoretical evidence for weaker correlations in electron-doped and Hg-based hole-doped cuprates

**DOI:** 10.1038/srep33397

**Published:** 2016-09-16

**Authors:** Seung Woo Jang, Hirofumi Sakakibara, Hiori Kino, Takao Kotani, Kazuhiko Kuroki, Myung Joon Han

**Affiliations:** 1 Department of Physics, Korea Advanced Institute of Science and Technology (KAIST), Daejeon 305-701, Korea; 2 Department of Applied Mathematics and Physics, Tottori University, Tottori 680-8552, Japan; 3 Computational Condensed Matter Physics Laboratory, RIKEN, Wako, Saitama 351-0198, Japan; 4 National Institute for Materials Science, Sengen 1-2-1, Tsukuba, Ibaraki 305-0047, Japan; 5 Department of Physics, Osaka University, Machikaneyama-Cho, Toyonaka, Osaka 560-0043, Japan; 6 KAIST Institute for the NanoCentury, Korea Advanced Institute of Science and Technology, Daejeon 305-701, Korea

## Abstract

Many important questions for high-*T*_*c*_ cuprates are closely related to the insulating nature of parent compounds. While there has been intensive discussion on this issue, all arguments rely strongly on, or are closely related to, the correlation strength of the materials. Clear understanding has been seriously hampered by the absence of a direct measure of this interaction, traditionally denoted by *U*. Here, we report a first-principles estimation of *U* for several different types of cuprates. The *U* values clearly increase as a function of the inverse bond distance between apical oxygen and copper. Our results show that the electron-doped cuprates are less correlated than their hole-doped counterparts, which supports the Slater picture rather than the Mott picture. Further, the *U* values significantly vary even among the hole-doped families. The correlation strengths of the Hg-cuprates are noticeably weaker than that of La_2_CuO_4_. Our results suggest that the strong correlation enough to induce Mott gap may not be a prerequisite for the high-*T*_*c*_ superconductivity.

Due to extensive efforts over the last 30 years^1^, significant progress has been made in the understanding of high-temperature superconducting materials. Although the pairing mechanism and the intriguing interplay between competing orders still remain elusive, many aspects of this series of copper-oxides have now been well established. Basically, all cuprates share common phase diagram features, and each phase has been a subject of intensive study. The ‘dome’-shaped region of superconductivity, which only appears after the long-range magnetic order is suppressed (see Fig. 1), is possibly the key to understanding the pairing principle of cuprates. These features are also found in other families of superconducting materials, such as Fe-based and heavy Fermion compounds, and have been well recognized, likely suggesting that the same superconducting mechanism exists in the different families^2^.

The superconducting dome has been considered to be particularly important in the framework of some outstanding theoretical models or ‘pictures’ that assume or predict its existence^3^,^4^. Therefore, it is striking that a series of recent experiments for electron-doped cuprates have reported data that contradicts this feature. According to a systematic re-investigation of electron-doped samples, RE_2_CuO_4_ (RE = rare-earth: Nd, Pr, Sm, etc.), the superconducting region does not cease to exist as the carrier concentration decreases, but this region extends to very low doping, quite close to zero^5^,^6^,^7^,^8^,^9^,^10^,^11^,^12^,^13^,^14^,^15^,^16^,^17^,^18^. Further, as the doping approaches zero, the superconducting transition temperature (*T*_*c*_) seems to keep increasing with no indication of the dome (see Fig. 1(b)). While further study needs to be performed to clarify this issue, it seems indicative that the undoped parent compounds of RE_2_CuO_4_ are a Slater-type insulator rather than a Mott-type insulator. Therefore, the ‘doped Mott insulator’ picture may not be appropriate, at least for the electron-doped family.

Some theoretical suggestions are supportive of this conclusion. According to Weber *et al.*^19^,^20^, for example, an electron-doped material, Nd_2_CuO_4_, is less correlated and should be identified as a Slater insulator, while the hole-doped La_2_CuO_4_ should be considered as a Mott insulator. The LDA + DMFT (local density approximation plus the dynamical mean field theory) calculation by Das and Saha-Dasgupta^21^ showed that the *T*-structured La_2_CuO_4_ is insulating while the *T*′-structured La_2_CuO_4_ is metallic at *U* = 4.5 eV. Comanac *et al.*^22^ also concluded that the correlation strengths in cuprates are not strong enough to be identified as Mott insulators.

In spite of its crucial importance, however, this issue is quite challenging because of the difficulty in quantifying the ‘Mott-ness’ or in estimating the correlation strengths. Here, we also note that while Comanac *et al.* concluded that all the cuprates are Slater insulators^22^, Weber *et al.*, as well as Das and Saha-Dasgupta, made a sharp distinction between the electron-doped and the hole-doped families^19^,^20^,^21^. One clear and well-defined way for resolving this issue is to calculate or ‘measure’ the material dependence of the correlation strength, which is traditionally denoted by the parameter *U* (on-site Coulomb repulsion within the single-band Hubbard model). Further, calculating the material dependent *U* values can illuminate other important issues such as pairing principle. Because electron-doped cuprates generally have lower *T*_*c*_ (≤30 K) than hole-doped materials, whose *T*_*c*_ sometimes exceeds 100 K (*e*.*g*., the triple-layered Hg-cuprates), it is important to determine if there is a notable difference in the correlation strengths of these two different families.

Here, we try to provide a clear answer to this long standing question by performing the direct estimation of *U* for several different types of cuprates. Our first-principles calculations show that both of the previous conclusions are not quite correct. On one hand, our result provides the first direct confirmation that the correlation strength of electron-doped materials is weaker than that of hole-doped counterparts. On the other, we significantly revise the previous conclusion: Not all of the hole-doped cuprates have stronger correlation compared to the electron doped ones. In fact, one representative hole-doped family, namely Hg-cuprates (and presumably many other multi-layered cuprates), has weaker electron correlation strength comparable to the electron-doped materials. Our result has a profound implication for the pairing principle: The correlation effects, strong enough to produce the Mott insulating state, may not be a prerequisite for high *T*_*c*_ superconductivity.

## Results

The results are summarized in Fig. 2. We clearly see that T’-structures (or, the parent compounds of electron-doped materials) have significantly smaller *U* values than the hole-doped materials (parent phases), especially La_2_CuO_4_. The calculated *U* for RE_2_CuO_4_ (RE: Nd, Pr, Sm) is 1.24–1.34 eV, which is considerably smaller than the La_2_CuO_4_ value of 3.15 eV. The material dependent *U*/*t* was also estimated (see Fig. 2; the data in green color and the right vertical axis), where the nearest-neighbor hopping parameter, *t*, was calculated with the standard Wannier-function technique^23^,^24^ (see Supplementary Information). The calculated *U*/*t* for La_2_CuO_4_ is ~7 which compares reasonably well with the widely used values for the model Hamiltonian studies^25^. The *U*/*t* value for the RE_2_CuO_4_ series is ~3, which is significantly smaller (~43% of the La_2_CuO_4_ value).

The 4*f* electrons in RE_2_CuO_4_ located around the Fermi level must be considered carefully. Because there is no well-established method to treat these states, first-principles calculations of rare-earth compounds has been challenging. One widely-used method is to treat the 4*f* electrons as part of the core electrons, as was done in refs 19 and 20. To minimize the ambiguity caused by this technical difficulty, we used three different methods; Method 1, 2, and 3 (see the Supplementary Information). For presentation, we took the average of these three values as the main data, and the error bars represent the largest and smallest values obtained by Methods 1–3 in Fig. 2. Importantly, our conclusions were the same regardless of which values are considered. In fact, if we consider the previously-used technique, Method 1, the *U*/*t* difference between the RE_2_CuO_4_ and La_2_CuO_4_ is enlarged (see the Supplementary Information).

Arguably, our calculation is the most direct way to determine the correlation strengths. For the estimation of correlation strength the previous theoretical approaches analyzed either the mass renormalization factor or the optical conductivity^19^,^20^,^21^,^22^ with *U* as a parameter. In the present study, we directly calculated *U* from first-principles without any adjustable parameter (see Methods and Supplementary Information). Therefore, our results, which show a smaller *U* value in electron-doped materials, can be regarded as direct evidence that materials with the *T*′-type lattice structure are less correlated.

**Figure 1:**
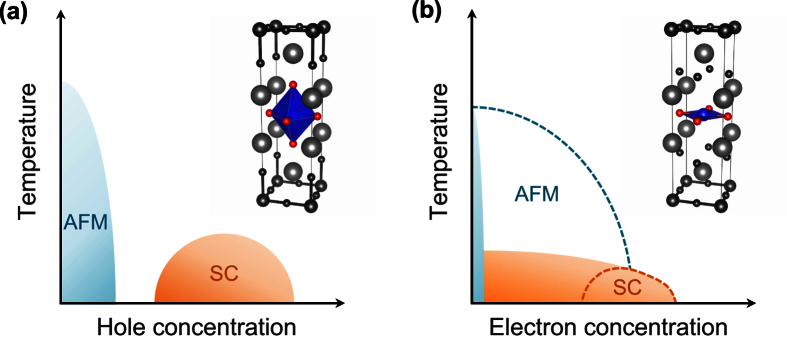
Schematic phase diagram of superconducting (SC) and antiferromagnetic (AFM) states for the (**a**) hole-doped and (**b**) electron-doped region. The insets show the representative crystal structure for each region: (**a**) La_2_CuO_4_ and (**b**) RE_2_CuO_4_ where the large, medium, and small spheres represent La/RE (grey), Cu (black or blue), and O (black or red), respectively. The octahedral CuO_6_ and planar CuO_4_ unit are shaded blue.

A characteristic feature that determines the material dependence of the correlation strength can be represented by a single parameter. Figure 3(a) shows the calculated *U*/*t* as a function of the inverse of the apical oxygen height (1/*h*_*O*_) (*i*.*e*., the average of the inverse bond distance between apical oxygen and copper). As 1/*h*_*O*_ increases, the increasing trend of *U*/*t* from the electron-doped materials, RE_2_CuO_4_, to the hole-doped HgBa_2_CuO_4_, and to La_2_CuO_4_ is obvious. For the case of RE_2_CuO_4_ with no apical oxygen, 1/*h*_*O*_ can be regarded as zero. While both (hole-doped) La_2_CuO_4_ and HgBa_2_CuO_4_ have well-defined octahedral oxygen cages around the Cu ions (*i*.*e*., CuO_6_), no apical oxygen is found in RE_2_CuO_4_, and CuO_4_ is formed instead of CuO_6_ (see Fig. 1, inset). The absence of two apical oxygen atoms can cause a significant difference in electronic properties and effectively reduce the correlation strengths. This relationship between *U*/*t* (or *U*) and *h*_*O*_ can be used as a good rule of thumb to measure the correlation strength.

**Figure 2:**
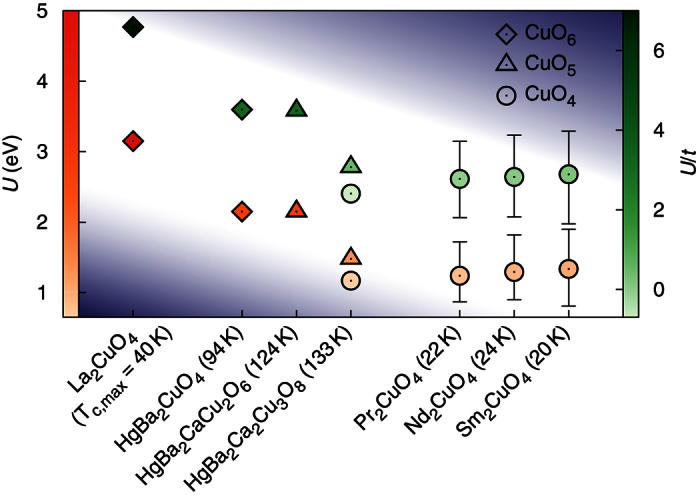
Calculated *U* and *U*/*t* for cuprate parent compounds. The left (orange) and the right (green) vertical axis correspond to *U* and *U*/*t*, respectively. A total of seven different materials have been calculated: La_2_CuO_4_ (single layered, hole doped), HgBa_2_CuO_4_ (single layered, hole doped), HgBa_2_CaCu_2_O_6_ (double layered, hole doped), HgBa_2_Ca_2_Cu_3_O_8_ (triple layered, hole doped), Pr_2_CuO_4_ (single layered, electron doped), Nd_2_CuO_4_ (single layered, electron doped), and Sm_2_CuO_4_ (single layered, electron doped). For the electron-doped materials, RE_2_CuO_4_, three different techniques have been used to treat the RE-4*f* electrons (see the text for more details). The average values are presented and the error bars indicate the largest and smallest values. The symbols represent the local CuO_*n*_ structures: diamonds, triangles, and circles correspond to CuO_6_, CuO_5_, and CuO_4_, respectively. The numbers in parentheses are the optimal superconducting *T*_*c*,max_ of each material.

**Figure 3:**
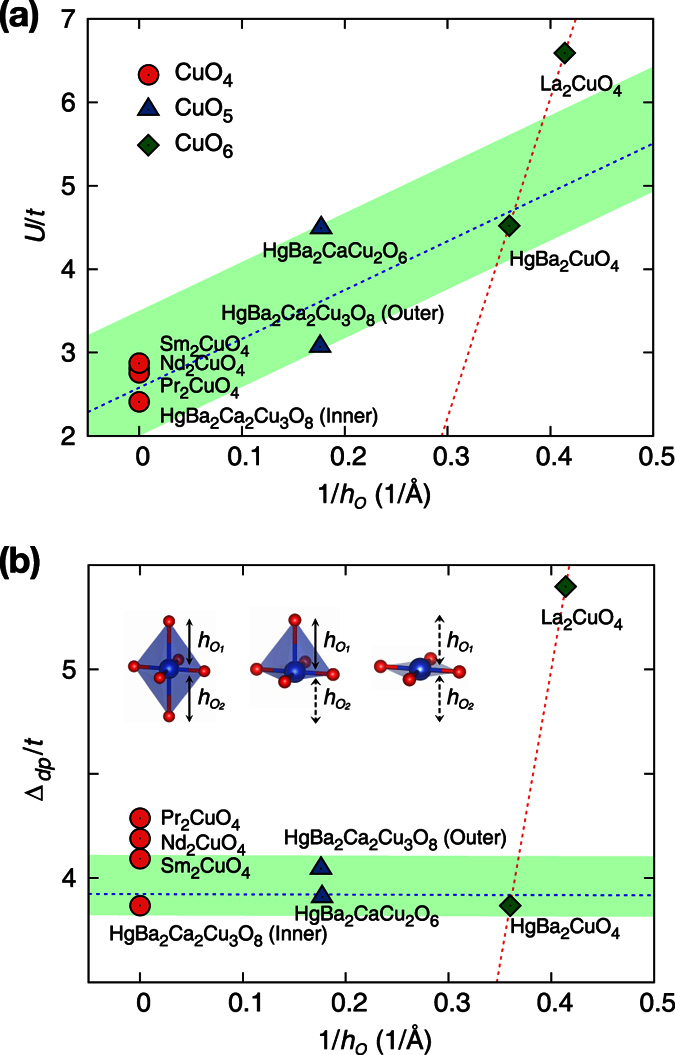
The calculated *U*/*t* (**a**) and Δ_*dp*_/*t* (**b**) as a function of the inverse apical oxygen height, 1/*h*_*O*_. The color and shape of each point represent the local structure of materials: CuO_6_ (green diamonds), CuO_5_ (blue triangles), and CuO_4_ (red circles) having two, one, and no apical oxygen, respectively. The local structures are presented in the inset of (**b**). The effective bond length between Cu and the apical oxygen, *h*_*O*_, is defined as 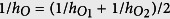 where 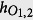 indicates the Cu to apical oxygen bond distance and the distance can be defined to be ∞ when there is no apical oxygen. For the case with no apical oxygen (CuO_4_), 1/*h*_*O*_ can be regarded as zero. For CuO_5_ which has one apical oxygen, 1/*h*_*O*_ is defined as half of the inverse of the bond distance between Cu and apical O. The red line shows the fitting from two data points of single-layer hole-doped compounds, La_2_CuO_4_ and HgBa_2_CuO_4_. The blue line shows the fitting from the four data points of the Hg-compounds. The shaded green blocks provide a guide for the eyes.

It is noteworthy that the hole-doped family can also have copper-oxygen layers with no apical oxygen. For example, the inner-layer of HgBa_2_Ca_2_Cu_3_O_8_ has the same local structure as RE_2_CuO_4_ (*i*.*e*., no apical oxygens; CuO_4_). Figures 2 and 3(a) clearly show that the inner-layer Cu in triple-layered HgBa_2_Ca_2_Cu_3_O_8_ has a similar value of *U* and *U*/*t* to RE_2_CuO_4_.

It is a remarkable new finding that some of the hole-doped cuprates have correlation strengths comparable to the electron-doped materials. It raises a question about the simple classification that categorizes all hole-doped cuprates as Mott insulators. As shown in Figs 2 and 3(a), the calculated *U* and *U*/*t* values of the Hg-cuprates are located in between those of RE_2_CuO_4_ and La_2_CuO_4_. Note that the single-layer HgBa_2_CuO_4_ has a well-defined CuO_6_ local unit as in La_2_CuO_4_, and its correlation strength is noticeably weaker than that of La_2_CuO_4_. According to our calculations, the difference of *U (U*/*t*) between HgBa_2_CuO_4_ and La_2_CuO_4_ is 1.0 eV (2.1). That difference is larger than the difference between HgBa_2_CuO_4_ and RE_2_CuO_4_, which is ~0.9 eV (~1.7). In the case of the triple-layer Hg-compounds, the correlation strengths decrease to be even closer to the values of electron-doped materials. We emphasize its significant implication for the pairing principle: Considering that the Hg-based cuprates exhibit quite high *T*_*c*_ ≥ 100 *K*, the correlation effects strong enough to produce the Mott insulating mother compound may not be a prerequisite for high *T*_*c*_ superconductivity.

It is instructive to see how these features are related to the charge transfer energy, Δ_*dp*_ = *E*_*d*_ (Cu-3*d* energy level) − *E*_*p*_ (O-2*p* energy level), which is another key parameter in many of the transition-metal oxides^26^. While Δ_*dp*_ is a quantity for the *d*-*p* model (not the single-band model), one can examine the behavior of Δ_*dp*_/*t* in comparison to *U*/*t*. Figure 3(b) shows the calculated Δ_*dp*_/*t* as a function of 1/*h*_*O*_. We note that the charge transfer energies of the Hg-compounds are more similar to the values of RE_2_CuO_4_ than those of La_2_CuO_4_. The overall behavior of *U* and Δ_*dp*_ is not quite different nor entirely similar. the same when plotted as a function of 1/*h*_*O*_. The similarity is likely due to that a large Δ_*dp*_ results in a smaller *d*-*p* hybridization, making Wannier orbital more localized. At the same time, the details of the band structure play some role in determining the correlation strength.

Importantly, the results of both *U* and Δ_*dp*_ indicate that Hg-compounds are significantly less correlated than La_2_CuO_4_, and their correlation strengths are comparable to those of electron-doped materials. Therefore, a simple classification of the parent compounds in terms of the carrier types is not pertinent, and the previous studies that regarded La_2_CuO_4_ as a prototype hole-doped cuprate should be re-interpreted. It may be more desirable to classify some of the hole-doped materials as Slater-type insulators.

## Discussion

Comparison of our result with experiments is not at all straightforward and any direct quantitative argument may not be possible. The determination of *U* based on any experimental data is eventually to fit onto a certain type of model. Within such an obvious limitation, it may be instructive to see the optical conductivity data as a possible consistency check. The previous experiments on the hole-doped materials, for example, seem basically consistent with our results: Charge transfer gap of La_2_CuO_4_ is larger than that of Nd_2_CuO_4_, and the integrated Drude weight of (doped) T’-materials is larger than La_2_CuO_4_. The trend of other materials is also compatible with our calculations while the data from the undoped parent compounds is not always available^22^,^27^,^28^,^29^,^30^,^31^,^32^.

Our results can provide natural explanations for recent experiments^7^,^8^,^9^,^10^,^11^,^12^,^13^,^14^,^15^,^16^ in which the phase diagram of the electron-doped cuprates exhibits monotonically increasing *T*_*c*_ toward zero doping (see Fig. 1(b)). This behavior has been observed in the carefully-annealed samples of both thin film and single crystal forms^7^,^8^,^9^,^10^,^11^,^12^,^13^,^14^,^15^,^16^. If it is indeed the case, the implication can be profound and the electron-doped side of the phase diagram should be re-drawn (Fig. 1(b)). According to our calculations, this behavior is a result of the relatively weak correlation in the electron-doped materials. In this context, it is instructive to recall a recent numerical result by variational Monte Carlo calculations. Yokoyama *et al.* showed in their one-band Hubbard model study that a small value of *U*/*t* ≤ 6 produces an increasing *T*_*c*_ region of superconductivity whereas a larger *U*/*t* value always gives the dome-shape^33^.

The Hg-cuprates are of interest in this regard. Being a hole-doped family, their correlation strength is significantly weaker than that of La_2_CuO_4_ and close to the electron-doped cuprates, especially in the triple-layer compound. Nevertheless, the dome-like doping dependence of *T*_*c*_ has been observed in both single-layer^34^ and multilayer^35^ Hg-cuprates. Therefore, the dome-shaped *T*_*c*_ may not necessarily be a consequence of strong electron correlation. In fact, a mechanism that can induce the dome-shaped *T*_*c*_ without Mott-ness has recently been proposed^36^. In this theory, the intrinsic electron-hole asymmetry of the hybridized Cu3*d*–O2*p* electronic structure plays an essential role. Regarding the absence or presence of antiferromagnetic ordering, it is important to note that the low doping regime (<5%) has not been experimentally reached for the single-layer Hg-compound due to the presence of excess oxygen^34^. Hence, considering the moderate value of *U*/*t* in single-layer Hg-cuprates, the presence of antiferromagnetism as well as the Mott-insulating state in the non-doping limit may still be an open issue. We expect the Tl-based cuprate, which also has a large *h*_*O*_ value, have similar behavior^37^. For multilayer Hg-compounds, antiferromagnetism has been reported in the underdoped regime^35^. Our result suggests that this insulating state can be of the Slater-type rather than the Mott-type. The robust presence of antiferromagnetism in these multilayer cases (compared to the electron-doped cases) might be due to the interlayer coupling.

## Summary and Conclusion

We performed the first direct calculation of the material dependent correlation strengths in cuprates. A clear increasing trend of *U* is found as a function of 1/*h*_*O*_. Our result strongly supports the Slater picture for electron-doped cuprates. It is the first direct evidence of weaker correlations in electron-doped materials, and can be regarded as a (theoretical) confirmation. On the other hand, we significantly revise the current understanding of this issue. Contrary to the previous conclusion, some of the hole-doped cuprates (*e*.*g*., the Hg-compounds) have considerably weaker correlations which are comparable to those in electron-doped materials. Our results indicate that the electron correlation strong enough to induce the Mott gap may not be a prerequisite for high *T*_*c*_ superconductivity.

## Methods

### Computation details

We used so-called ‘constrained random phase approximation (cRPA)’ method to estimate the correlation strength. This recently-established technique^38^,^39^,^40^,^41^,^42^,^43^,^44^,^45^,^46^ has been proven to be reliable in many different types of materials^40^,^41^,^42^,^43^,^44^,^45^,^46^,^47^,^48^,^49^,^50^,^51^,^52^,^53^,^54^,^55^,^56^, including 3*d*, 4*d*, 5*d* transition-metal oxides^47^,^48^,^49^,^50^,^51^,^52^ and Fe-based superconductors^53^,^54^,^55^,^56^, while it has never been systematically applied to cuprates. Early calculations of La_2_CuO_4_ based on constrained LDA (cLDA) predict too large *U* value of ~7–10 eV^57^,^58^,^59^,^60^,^61^. It is a typical feature of cLDA due to the limitation for describing the electronic screening^41^. Our implementation of cRPA into our own software package ‘*ecalj*’^62^ follows one of the most recent standard formalisms by Şaşιoğlu *et al.*^44^,^45^ (see the Supplementary Information). We have checked that the previously reported data for many different materials were well reproduced by our implementation (see the Supplementary Information).

In order to avoid the ambiguity related to the 4*f* electrons in RE_2_CuO_4_, we used three different methods. Method 1 treats the RE-4*f* orbitals as the core as in the previous studies^19^,^20^. This method removes some screening channels (but not the on-site *d*-*d* transitions) around the Fermi energy and can cause some deviation in the *U* estimation. Method 2 replaces RE ions with La while maintaining the experimental lattice parameters. The resulting effect is expected to be similar to Method 1. We emphasize, however, that the whole procedure is determined in a self-consistent way, and the position and the width of the Cu-3*d* band is adjusted accordingly. Therefore, the naive guess for the final *U* value might not be correct. Method 3 keeps the RE-4*f* states around the Fermi energy as described by LDA. Within LDA, these less-renormalized and uncorrelated 4*f*-bands are located closer to the Fermi level and contribute to the screening. In spite of the complexity of the LDA band structure, the Cu-*e*_*g*_ bands are well identified by the standard Wannier fitting, and therefore, Method 3 works as well as the other two approaches (see the Supplementary Information). The average of these three values is presented as the main data while the error bars represent the largest and smallest values obtained by Methods 1–3 (Fig. 2).

The LDA band structure was calculated by an all-electron full-potential method with the PMT basis (augmented plane wave + muffin-tin orbital)^63^. The polarization function is expanded by the mixed product basis in which the imaginary part along the real axis is accumulated with the tetrahedron method and the real part is obtained by a Hilbert transformation. Our approach has a clear advantage in terms of its accuracy compared to other methods, such as simple **k**-point sampling, Matsubara-frequency sampling, and the pseudopotential method. We have carefully verified the **k**-point dependency and found that our conclusions are robust against the computation details (see the Supplementary Information). The calculated *U* value of 3.15 eV for La_2_CuO_4_ is in good agreement with the only available data of 3.65 eV^49^. For further details, see the Supplementary Information.

## Additional Information

**How to cite this article**: Jang, S. W. *et al.* Direct theoretical evidence for weaker correlations in electron-doped and Hg-based hole-doped cuprates. *Sci. Rep.* **6**, 33397; doi: 10.1038/srep33397 (2016).

## Supplementary Material

Supplementary Information
