# Supplementary material for: Direct theoretical evidence for weaker correlations in electron-doped and Hg-based hole-doped cuprates
Source: Sci Rep. 2016 Sep 16;6:33397. doi: 10.1038/srep33397 (PMC5025755; doi:10.1038/srep33397)
Supplement: Supplementary Information [file srep33397-s1.pdf]

Supplementary information:
Direct theoretical evidence for weaker correlations in electron-doped and Hg-based hole-doped cuprates

Seung Woo Jang,[1] Hirofumi Sakakibara,[2] Hiori Kino,[3] Takao Kotani,[4] Kazuhiko Kuroki,[5] and Myung Joon Han[1,6,*]

[1]*Department of Physics, Korea Advanced Institute of Science and Technology (KAIST), Daejeon 305-701, Korea*

[2]*Computational Condensed Matter Physics Laboratory, RIKEN, Wako, Saitama 351-0198, Japan*

[3]*National Institute for Materials Science, Sengen 1-2-1, Tsukuba, Ibaraki 305-0047, Japan.*

[4]*Department of Applied Mathematics and Physics, Tottori University, Tottori 680-8552, Japan*

[5]*Department of Physics, Osaka University, Machikaneyama-Cho, Toyonaka, Osaka 560-0043, Japan*

[6] *KAIST Institute for the NanoCentury, Korea Advanced Institute of Science and Technology, Daejeon 305-701, Korea*

*Electronic address: `mj.han@kaist.ac.kr`

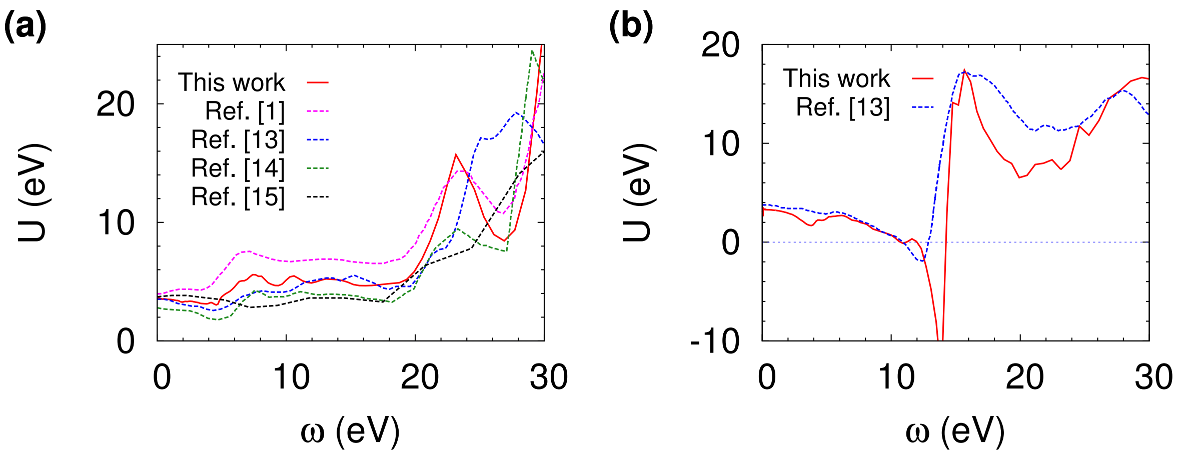

FIG. S1: The calculated $U(\omega)$ (red solid lines) for (a) paramagnetic Ni and (b) $SrVO_3$ in comparison with the previous studies (dashed lines). The five $3d$ states and the three t_{2g} states are averaged for (a) Ni and (b) $SrVO_3$, respectively.

TABLE S1: The calculated values of $U(\omega = 0)$ for paramagnetic Ni and $SrVO_3$

	U (eV)					
Material	This work	Ref. [13]	Ref. [14]	Ref. [15]	Ref. [1]	Ref. [16]
Ni	3.56	3.7	2.77	3.40–4.05[a]	3.96	N/A
$SrVO_3$	3.15	3.5	3.0	N/A	N/A	3.2

[a]Miyake *et al.* [15] showed that the static U can be slightly varied with the different energy windows chosen for Wannier functions.

I. COMPUTATION DETAILS

We implemented a recent cRPA formalism refined by Şaşıoğlu *et al.* [1–3] in a first-principles electronic structure calculation package '`ecalj`' [4, 5], which is originally designed for quasiparticle self-consistent GW calculations [6]. Our method has computational advantage by keeping the positive definiteness of the imaginary part of screened Coulomb interaction; the same technique with that of Ref.[7]. In all calculations, we used the experimental crystal structures [8–12]. The interaction parameter, $U_{x^2-y^2}$, is estimated by both one band fitting and two band (e_g) fitting, and the results are in good agreement with each

other. The most time-consuming check procedure is about the **k**-point convergence. We have carefully performed this check (see Section II in the below). The number of **k** points used in cRPA calculations are 8×8×8, 8×8×4, 8×8×3, 8×8×2, 8×8×8 for the first Brillouin zone of La_2CuO_4, $HgBa_2CuO_4$, $HgBa_2CaCu_2O_6$, $HgBa_2Ca_2Cu_3O_8$, and RE_2CuO_4 respectively. For LDA calculations, the number of **k** points were increased; 12×12×12, 12×12×8, 12×12×4, 12×12×4, and 12×12×12 for La_2CuO_4, $HgBa_2CuO_4$, $HgBa_2CaCu_2O_6$, $HgBa_2Ca_2Cu_3O_8$, and RE_2CuO_4, respectively. Since the **k**-points for the multilayer Hg-compounds could not be as many as for the others due to the large computational cost, our results for these cases could be slightly overestimated (see Section II). However, it will not affect any of our conclusions as clearly seen in Section II. Our code has been tested with paramagnetic Ni and $SrVO_3$ which are the most extensively-examined systems in the literature [1, 13–16]. As shown in Table S1 and Fig. S1, our results are in good agreement with the previous ones.

II. K-POINT TEST AND OTHER NUMERICAL DETAILS

We have carefully checked the **k**-point dependence of $U(\omega)$ for all materials considered in this study. The systematic behaviors are always found as the number of **k**-points increases as shown in Fig. S2 where we chose La_2CuO_4 (a), $HgBa_2CuO_4$ (b) and Pr_2CuO_4 (Method 3) (c) as the representative examples. This test calculation clearly shows that the **k** meshes we used is good enough with only two exceptions of $HgBa_2CaCu_2O_6$ and $HgBa_2Ca_2Cu_3O_8$. As discussed in the main text, the **k** meshes used for these multilayer Hg-cuprates may not be enough and the U values get slightly reduced by increasing the **k** points. However our **k**-point test shows that the change would not be significant (presumably ∼0.1 eV) and our conclusions not be changed.

We paid special attention to the low frequency oscillation of $U(\omega)$ (Fig.S3). They are observed in our implementation most likely due to the incomplete matching between the original d and Wannier bands [1–3] although such low energy excitations should in principle be absent within the original spirit of cRPA. The size and the position of remnant excitations are related to the discrete **k** sum around Fermi surface, and they become less pronounced as we use larger number of **k** points (Fig.S3). Also from the other check calculations based on Kramers-Kronig relation, we conclude that the error caused by this excitation is just about ∼0.1 eV.

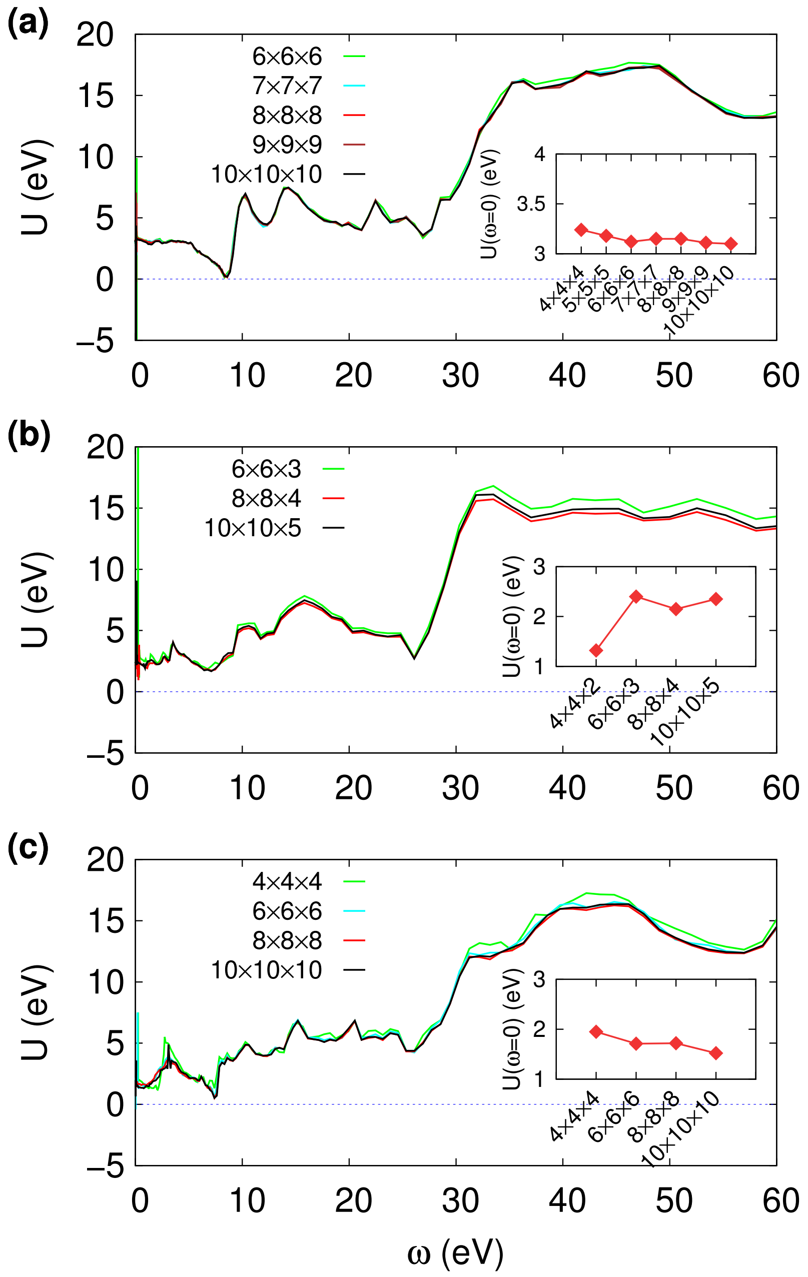

FIG. S2: The calculated $U(\omega)$ with different numbers of $\mathbf{k}$ points for (a) La_2CuO_4, (b) $HgBa_2CuO_4$, and (c) Pr_2CuO_4 (Method 3). The insets show the calculated $U(\omega = 0)$ corresponding to each $\mathbf{k}$ mesh.

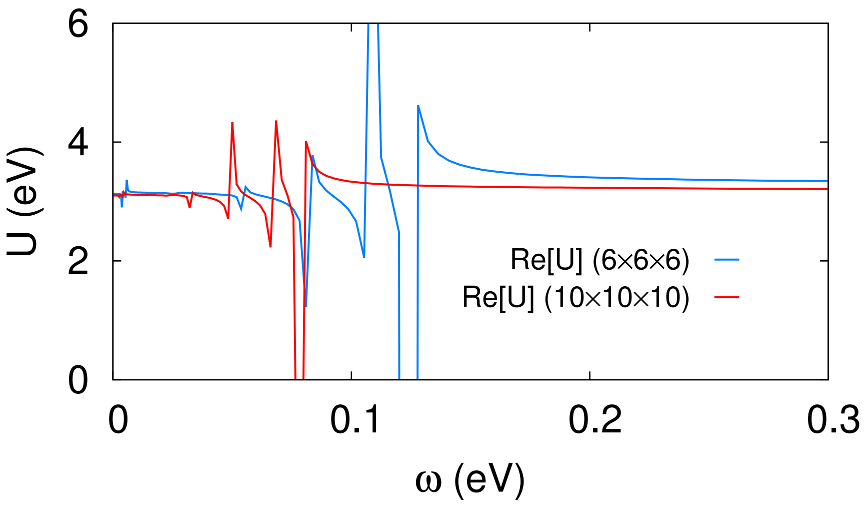

FIG. S3: The calculated $U(\omega)$ of La_2CuO_4 in the low frequency regime. The blue and red line represents the results from 6×6×6 and 10×10×10 **k**-mesh, respectively. The oscillation due to the remnantal screening becomes less significant as the number of **k** points increases and at the lower frequency. The error inevitably caused by this kind of oscillation is expected to be ∼0.1 eV (see the text for more details).

III. WANNIER FITTING

We have performed the Wannier fit by considering both the $d_{x^2-y^2}$ and d_{z^2} orbitals explicitly as the internal space [17], and here we present only the $d_{x^2-y^2}$ bands, for which U was estimated. The Wannier band fitting works well for all cases including the multilayer Hg-cuprates (having multiple Cu-$d_{x^2-y^2}$ bands) and the RE_2CuO_4 (having RE-4f states around Fermi energy; Method 3). The results are presented in Figure S4 and S5.

IV. MODEL PARAMETERS

The calculation results for t, t', U, U/t, Δ_{dp}, and J are summarized in Table S2. The Δ_{dp} calculated by Weber *et al.* [18] is also presented for comparison.

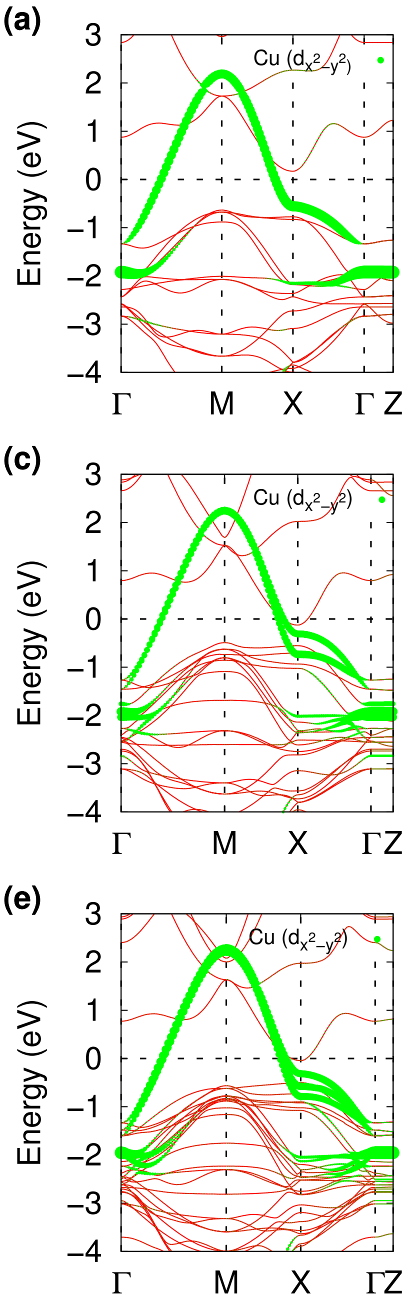

FIG. S4: The calculated LDA band structure (a, c, e) and the Wannier band (b, d, f) for Hg-compounds. The single-, double-, and triple-layer cases are presented in (a, b), (c, d), and (e, f), respectively. In (a, c, e), the Cu-$d_{x^2-y^2}$ character is represented by the green color. In (b, d, f), the $d_{x^2-y^2}$ Wannier band is depicted by the blue cross on top of the calculated band dispersion (red lines).

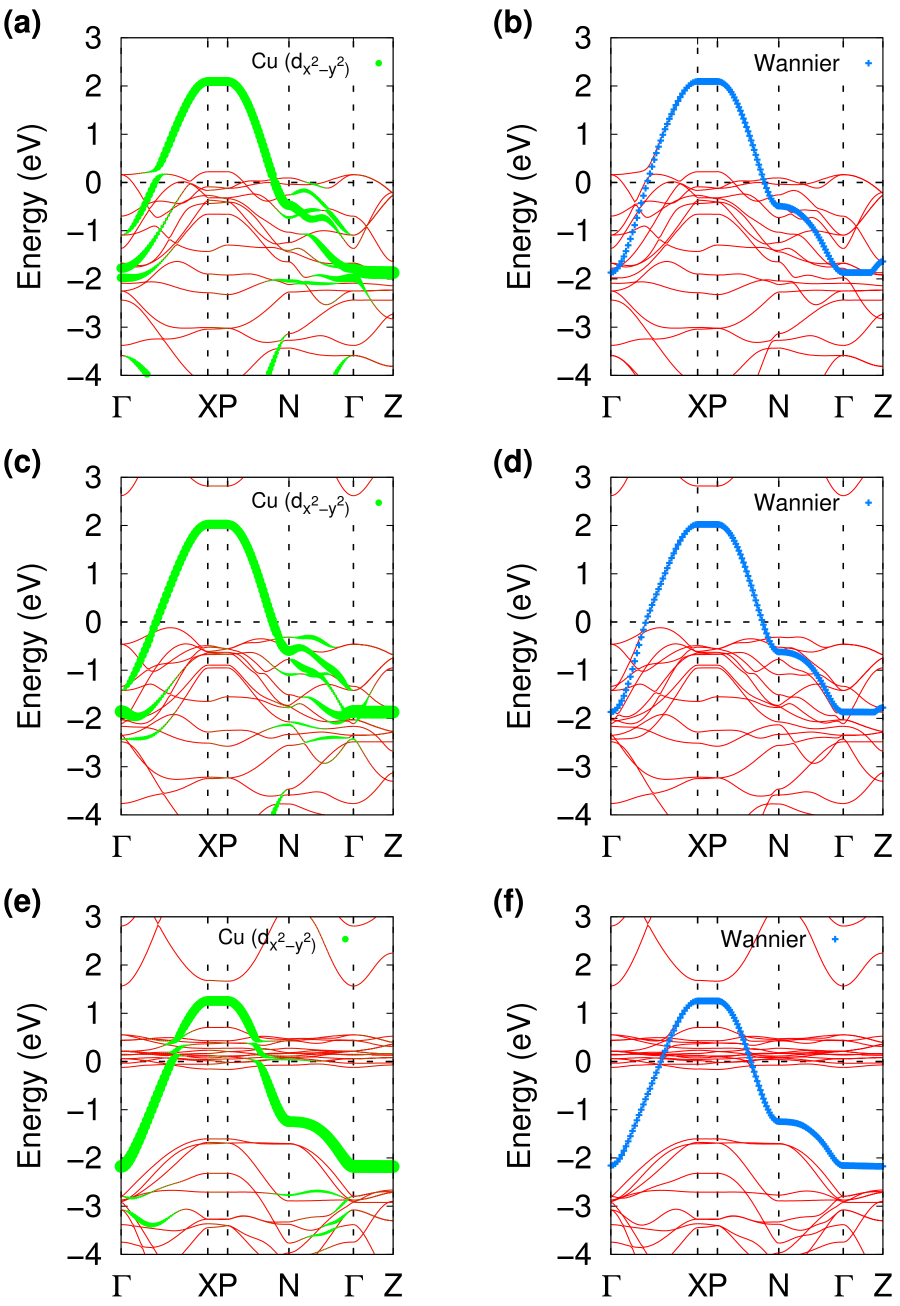

FIG. S5: The calculated LDA band structure (a, c, e) and the Wannier band (b, d, f) of Pr_2CuO_4. The results from Method 1, 2, and 3 are presented in (a, b), (c, d), and (e, f), respectively. In (a, c, e), the Cu-$d_{x^2-y^2}$ character is represented by the green color. In (b, d, f), the $d_{x^2-y^2}$ Wannier band is depicted by the blue cross on top of the calculated band dispersion (red lines).

Material	t (eV)	t' (eV)	U (eV)	U/t	Δ_{dp} (eV)	Δ_{dp} (Ref.[18]) (eV)	J (eV)
La_2CuO_4	0.478	0.096	3.15	6.59	2.58	2.76	0.43
$HgBa_2CuO_4$	0.476	0.095	2.15	4.52	1.84	N/A	0.58
$HgBa_2CaCu_2O_6$	0.479	0.106	2.15	4.49	1.87	N/A	0.49
$HgBa_2Ca_2Cu_3O_8$ (Outer-layer)	0.482	0.106	1.48	3.07	1.95	N/A	0.25
$HgBa_2Ca_2Cu_3O_8$ (Inner-layer)	0.486	0.107	1.17	2.41	1.88	N/A	0.33
Pr_2CuO_4 (Method 1)	0.483	0.088	0.87	1.80	1.37	1.65	0.39
Pr_2CuO_4 (Method 2)	0.462	0.090	1.72	3.72	1.66	N/A	0.52
Pr_2CuO_4 (Method 3)	0.403	0.108	1.13	2.80	2.59	N/A	0.52
Nd_2CuO_4 (Method 1)	0.493	0.088	0.90	1.83	1.39	1.61	0.35
Nd_2CuO_4 (Method 2)	0.470	0.093	1.82	3.87	1.72	N/A	0.52
Nd_2CuO_4 (Method 3)	0.417	0.109	1.16	2.78	2.54	N/A	0.53
Sm_2CuO_4 (Method 1)	0.491	0.091	0.81	1.65	1.42	N/A	0.31
Sm_2CuO_4 (Method 2)	0.478	0.095	1.90	3.97	1.76	N/A	0.52
Sm_2CuO_4 (Method 3)	0.426	0.110	1.30	3.05	2.43	N/A	0.52

TABLE S2: The summary of the calculated model parameters.

[1] E. Şaşıoğlu, C. Friedrich, and S. Blügel, Phys. Rev. B **83**, 121101(R) (2011).

[2] E. Şaşıoğlu, C. Friedrich, and S. Blügel, Phys. Rev. Lett. **109**, 146401 (2012).

[3] E. Şaşıoğlu, I. Galanakis, C. Friedrich, and S. Blügel, Phys. Rev. B **88**, 134402 (2013).

[4] The electronic structure simulation package, "ecalj". https://github.com/tkotani/ecalj whose one-body part is developed based on Ref.[5].

[5] LMTO electronic structure simulation package, "LM Suite". http://www.lmsuite.org whose *GW* part is adopted mainly from Ref.[4].

[6] T. Kotani, J. Phys. Soc. Jpn. **83**, 094711 (2015).

[7] T. Kotani, J. Phys.: Condens. Matter **12**, 2413 (2000).

[8] J. D. Jorgensen, H. -B. Schuttler, D. G. Hinks, D. W. Capone, K. Zhang, M. B. Brodsky, and D. J. Scalapino, Phys. Rev. Lett. **58**, 1024 (1987).

[9] J. L. Wagner, P. G. Radaelli, D. G. Hinks, J. D. Jorgensen, J. F. Mitchell, B. Dabrowski, G. S. Knapp, and M. A. Beno, Physica C **210**, 447 (1993).

[10] M. Cantoni, A. Schilling, H. -U. Nissen, and H. R. Ott, Physica C **215**, 11 (1993).

[11] M. S. Kaluzhskikh, S. M. Kazakov, G. N. Mazo, S. Y. Istomin, E. V. Antipov, A. A. Gippius, Y. Fedotov, S. I. Bredikhin, Y. Liu, G. Svensson, and Z. Shen, J. Solid State Chem. **184**, 698 (2011).

[12] T. Chattopadhyay, P. J. Brown, and U. Köbler, Physica C **177**, 294 (1991).

[13] F. Aryasetiawan, K. Karlsson, O. Jepsen, and U. Schönberger, Phys. Rev. B **74**, 125106 (2006).

[14] T. Miyake and F. Aryasetiawan, Phys. Rev. B **77**, 085122 (2008).

[15] T. Miyake, F. Aryasetiawan, and M. Imada, Phys. Rev. B **80**, 155134 (2009).

[16] L. Vaugier, H. Jiang, and S. Biermann, Phys. Rev. B **86**, 165105 (2012).

[17] H. Sakakibara, H. Usui, K. Kuroki, R. Arita, and H. Aoki, Phys. Rev. Lett. **105**, 057003 (2010).

[18] C. Weber, K. Haule, and G. Kotliar, Phys. Rev. B **82**, 125107 (2010).
